# Toxicity of anthelmintic drugs (fenbendazole and flubendazole) to aquatic organisms

**DOI:** 10.1007/s11356-014-3497-0

**Published:** 2014-09-06

**Authors:** Marta Wagil, Anna Białk-Bielińska, Alan Puckowski, Katarzyna Wychodnik, Joanna Maszkowska, Ewa Mulkiewicz, Jolanta Kumirska, Piotr Stepnowski, Stefan Stolte

**Affiliations:** 1 Department of Environmental Analysis, Faculty of Chemistry, University of Gdańsk, ul. Wita Stwosza 63, 80-308 Gdańsk, Poland; 2 UFT Center for Environmental Research and Sustainable Technology, University of Bremen, Leobener Straße, D-28359 Bremen, Germany

**Keywords:** Ecotoxicity, Flubendazole, Fenbendazole, Aquatic species, Anthelmintic drugs, Benzimidazoles

## Abstract

Flubendazole (FLU) and fenbendazole (FEN) belong to benzimidazoles—pharmaceuticals widely used in veterinary and human medicine for the treatment of intestinal parasites as well as for the treatment of systemic worm infections. In recent years, usage of these drugs increased, which resulted in a larger contamination of the environment and possible negative effects on biota. Hence, in our research, we investigated an aquatic ecotoxicity of these pharmaceuticals towards: marine bacteria (*Vibrio fischeri*), green algae (*Scenedesmus vacuolatus*), duckweed (*Lemna minor*) and crustacean (*Daphnia magna*). Ecotoxicity tests were combined with chemical analysis in order to investigate the actual exposure concentration of the compounds used in the experiment as well as to stability and adsorption studies. As a result, study evaluating sensitivity of different aquatic organisms to these compounds and new ecotoxicological data is presented. The strongest negative impact of FLU and FEN was observed to *D. magna*.

## Introduction

Flubendazole (FLU) and fenbendazole (FEN)—belonging to benzimidazoles group—are anthelmintic drugs widely used in veterinary medicine in order to treat diseases in agriculture and aquaculture and also in human medicine (Danaher et al. 2006). Being excreted from the body with faeces and urine, they reach environment via different routes. According to literature, residues of FLU were found in the leachate from agricultural manure to drainage waters reaching values of up to 300 ng L^−1^ (Weiss et al. 2008) as well as in influent (19.9–89.7 μg L^−1^) and effluent (55.0–671.0 ng L^−1^) wastewater from the pharmaceutical industry (Van De Steene and Lambert 2008). Moreover, they were also detected in the surface waters (the Llobregat River, Spain) at the concentrations up to 1.32 ng L^−1^ (Zrnčić et al. 2014). Hence, these compounds as well as other pharmaceuticals have been classified as emerging environmental contaminants for almost 15 years now. Their structural formulas and selected physicochemical properties are presented in Table 1. Special attention should be paid to the high values of octanol–water partition coefficients of FLU (2.91 (Horvat et al. 2012)) and FEN (3.93 (Mottier et al. 2003)) which can influence their environmental fate and bioavailability. These chemicals are designed to have a specific mode of action which is binding to β-tubulin and inhibition of microtubule formation in the intestinal cells inducing a decreased glucose uptake and starving of the parasites (Martin 1997). Since microtubules serve a variety of important functions in animal, plant, fungi and some bacterial cells make FLU and FEN to be evaluated for potential effects on aquatic flora and fauna. Even though the health-risk assessment of pharmaceutical compounds regarding their toxicity is available, little is known about the ecotoxicological effects on non-target organisms. Toxicity of many other pharmaceuticals (with special emphasis to veterinary drugs) has been demonstrated in various aquatic organisms (e.g.: Tišler and Kožuh Eržen 2006; Park and Choi 2008; Santos et al. 2010; Białk-Bielińska et al. 2011; Kołodziejska et al. 2013).

**Table 1:** Structural formulas and selected physicochemical properties of flubendazole (FLU) and fenbendazole (FEN)

Compound	Chemical structure	Molecular weight [g mol^−1^]	log *K*_ow_	pK_a_	Water solubility [mg L^−1^]
FLU		313.3	2.91^a^	3.6, 9.6^a^	<10^b^
FEN		299.4	3.93^c^	5.12, 12.72^d^	0.01–0.04^a^

^a^Horvat et al. 2012

^b^Nobilis et al. 2007

^c^Mottier et al. 2003

^d^Santana Rodríguez et al. 2010

Aquatic organisms are particularly important targets, as they are exposed via wastewater residues over their whole life. Once inside the organism, the pollutant may promote a variety of effects, ranging from cellular impairment to lethality. As Escher has highlighted the consideration of underlying molecular mechanisms and modes of toxic action on different levels of biological organization is crucial to enhance the understanding of the effects of pollutants on living systems (Escher 2001). However, such studies are very limited.

There are only few literature data on the ecotoxicity of FLU and FEN towards limited number of aquatic species. From the species selected to our study, this includes only studies on *Daphnia magna* and *Vibrio fischeri* (Hoechst-Roussel Agri-Vet 1995; Oh et al. 2006). Moreover, these ecotoxicity tests have not been performed in combination with chemical analysis, which according to Garcia-Galán et al., enables to acquire a complete view of the exposure and the risk posed by these pollutants by the simple determination the chemical composition of the sample (García-Galán et al. 2009). Generally, there is no data available on the toxicity of FLU and FEN to green algae as well as to duckweed.

For all the above-mentioned reasons, the main aim of this study was to evaluate the ecotoxicity of FEN and FLU towards four aquatic organisms representing different levels of biological organization: luminescent marine bacteria (*Vibrio  fischeri*), limnic unicellular green algae (*Scenedesmus vacuolatus*), duckweed (*Lemna minor*) and crustacean (*Daphnia  magna*). This aim was achieved by the following:

- By performing ecotoxicological tests in combination with instrumental analysis which not only increase the reliability of the obtained ecotoxicological data but also enables to acquire a complete view of the exposure (bioavailability) and hence the risk posed by the pollutants. This aspect is very crucial as, due to the different physicochemical properties of chemicals (such as water solubility), their nominal concentration in the tests can essentially differ from the real one, which leads to underestimation of the hazard posed by the tested substances.
- By performing additional chemical studies and calculations in the case of the most sensitive organism (*D. magna*). These experiments were aimed at better understating of the reason for the observed toxicity. The investigation included determining the fate of the substance during the test conditions (stability and adsorption studies), as well as distinguishing the specific or non-specific mode of toxic action of these compounds by applying the baseline toxicity model. The reason for this is the fact that the observed toxicity of a chemical is not necessarily associated with its parent form, but rather its degradation products—which can be produced under the test conditions (e.g. by hydrolysis or photolysis) and can be even more toxic. Moreover, also adsorption of chemicals on tests vessels is very important, as it can decrease the concentration of the chemical during the test, hence lowering the toxicity.

Although such studies are nowadays recommended, they are still limited.

## Materials and methods

All the data presented in our study were obtained according to internationally/nationally accepted test guidelines (e.g. OECD, ISO, DIN) or their modified versions.

### Chemicals

FLU and FEN were purchased from Santa Cruz Biotechnology Inc. (Heidelberg, Germany). Acetonitrile (ACN), dimethyl sulfoxide (DMSO) and salts used for preparing culture media were supplied from Sigma-Aldrich (Steinheim, Germany).

### Standard stock solutions

The standard stock solutions of FLU and FEN (500 mg L^−1^) were prepared separately by dissolving them in DMSO. These solutions were stored at above 18 °C (melting point of DMSO), in a dark and dry place. Stock solutions for ecotoxicity testing were prepared by dissolving standard stock solution in media solutions used in each ecotoxicity test.

### Luminescent inhibition assay with marine bacteria

The toxicity test based on *V. fischeri* was done using the LCK 482 test kit (Dr Lange GmbH, Germany). The 30-min standard bioluminescence inhibition assay was carried out according to a modified DIN 38412-L34 protocol (1991). The tests were carried out three times for each substance with two parallel replicates in each test. At least four organic solvent-free controls (containing only medium: 2 % NaCl solution, phosphate-buffered) and two solvent controls (containing 0.06 % DMSO in medium) were used during each test. The tests were performed at 15 °C using thermostats (LUMIStherm, Dr Lange GmbH, Germany). The luminescence was measured with a luminometer (LUMIStox 300, Dr Lange GmbH, Germany). The freeze-dried bacteria were rehydrated according to the test protocol; then, 500 μL aliquots of the bacteria solution were pre-incubated for 15 min at 15 °C. After the initial luminescence had been measured, 500 μL of the diluted samples were added. The bioluminescence was measured again after an incubation time of 30 min. The relative toxicity of the samples was expressed as a percentage inhibition compared to the controls.

### Reproduction inhibition assay with limnic green algae *S. vacuolatus*

A synchronized culture of the green algae *S. vacuolatus* (strain 211–15, SAG (Culture Collection of Algae), Universität Göttingen, Germany) was used for this assay. The stock culture was grown under photoautotrophic conditions at 28 °C (±0.5 °C) in an inorganic, sterile medium (pH 6.4) with saturating white light (22 to 33 klx, Lumilux Daylight L 36 W-11 and Lumilux Interna L 36 W-41, Osram, Berlin, Germany). The cells were aerated with 1.5 vol% CO_2_ and synchronized using a 14- to 10-h light–darkness cycle. The stock culture was diluted every day to a cell density of 5 × 10^5^ cells mL^−1^. This test is a modified version of the assay described by Altenburger et al. (1990), and its sensitivity is comparable to the standardized 72-h test (ISO Guideline 8692 1989).

The toxicity tests started with autospores. The algae were exposed to the test substances for one growth cycle (24 h). The endpoint of this assay is the inhibition of algal reproduction, measured as the inhibition of population growth. Cell numbers were determined with a Coulter Counter Z2 (Beckmann, Nürnberg, Germany). The tests were performed in sterilized glass tubes, the algae were stirred throughout the 24-h test period, and the test conditions were the same as for the stock culture except for the CO_2_ source. Here, 150 μL of NaHCO_3_ solution was added to each test tube. The methods of stock culturing and testing are described in detail by Faust et al. (2001). Growth inhibition was calculated using the cell counts of the treated samples in relation to the untreated controls. Six organic solvent-free controls (containing only medium) and six solvent controls (containing 0.2 % DMSO in medium) were used for each assay. The tests were performed using six different concentrations in two replicates of every compound and each test was repeated three times.

### Growth inhibition assay with duckweed

The growth inhibition assay with *L. minor* was performed according to a modified version of the test protocol described in detail by Drost et al. (2007). The plants were grown in open Erlenmeyer flasks in sterilized Steinberg medium (pH 5.5 ± 0.2) in a climate chamber with a constant temperature of 25 ± 2 °C. To exclude pH effects on plant growth, the pH was checked at the beginning and end of the test. Based on control sample evaluation, the pH changes did not affect growth inhibition. The chamber was illuminated continuously with a maximum of 6 klx. The assays were performed on six-well cell culture plates (Greiner Bio-One GmbH, Frickenhausen, Germany). The tests were performed using six different concentrations in three replicates of every compound and each test was repeated three times. Six organic solvent free controls (containing only medium) and six solvent controls (containing 0.2 % DMSO in medium) were used in each test. The test started with one plant consisting of three duckweed fronds, and the measured endpoint was the inhibition of the growth rate determined by the frond area (mm^2^), which was calculated for the treated plants in relation to the untreated controls. The frond area was detected using a Scanalyzer from Lemnatec GmbH (Würselen, Germany).

### Growth inhibition assay with *D. magna*

The 48-h acute immobilization test with *D. magna* was assessed using the commercially available Daphtoxkit F (MicroBioTest Incorporation, Gent, Belgium), referred to OECD guideline 202 (2004). The detailed description of this assay is given in the supplier’s standard operational procedure (MicroBioTest Inc and Daphtoxkit 1996). The tests with neonates less than 24-h old, obtained by the hatching of ephippia, were performed at 20 °C in the dark. Five pre-fed animals were incubated with the toxicants in a volume of 10 mL of mineral medium in the plastic plates. In each test, five different concentrations of the test substance were investigated in five parallels and five solvent-free controls (medium) as well as five solvent controls (containing 0.015 % DMSO in medium for FLU and 0.01 % DMSO in medium for FEN). All the experiments were performed in two parallel replicates and repeated twice. The numbers of immobilized organisms were checked after 24 and 48 h. The sensitivity of the organisms to K_2_Cr_2_O_7_ was checked routinely once a new batch of organisms was obtained.

### Effect data modeling

Dose–response curve parameters and plots were obtained using the drift package (version 0.05-95) for the R language and environment for statistical computing (www.r-project.org).

### Instrumental analysis

In order to determine the soluble fraction of the investigated compound in biological media, HPLC–DAD analysis was performed. VWR Hitachi HPLC–DAD systems (containing the L-2130 HTA-pump, L-2130 degasser, L-2200 autosampler, L-2300 column oven, L-2450 diode array-detector and the EZChrom Elite software, VWR, Darmstadt, Germany) was used. The separation was performed on Gemini C_18_ column (150 × 4.6 mm I.D., 5 μm pore size, Phenomenex Inc., Torrance, CA). A mixture of A–ACN and B–H_2_O was used as a mobile phase (gradient conditions, 30 % A in 0 min, 60 % A in 5 min and 30 % A in 10 min). The injection volume of the sample was 50 μL. A detection wavelength of 300 nm was used for the quantification. The analysis time was 15 min.

The concentrations of stock solution of FLU and FEN in media solutions used in four ecotoxicity tests were as follows: 1 mg L^−1^ for *L. minor* and *S. vacuolatus* (the solution consisted 0.2 % DMSO), 0.05 mg L^−1^ (0.01 % DMSO) for FEN and 0.075 mg L^−1^ (0.015 % DMSO) for FLU for *D. magna* and 0.3 mg L^−1^ for *V. fischeri* (0.06 % DMSO). In order to calculate the bioavailability, the peak area of the signals obtained from the analysis of the above-mentioned mixtures were compared to the peak area of the signals obtained from the analysis of the same concentrations of FLU and FEN prepared in ACN.

### Chemical stability and adsorption studies

As strong negative effect of the tested compounds was observed on *D. magna* (see “Toxicity tests”) additional experiments (chemical stability and adsorption studies) were performed. These assays were performed in two types of vessels: in a plastic (polycarbonate) and a glass beaker. Each vessel was filled with 10 mL of the substance solution prepared in *D. magna* test medium. For FEN and FLU, the concentration used in this test was 0.05 and 0.075 mg L^−1^, respectively, which was the highest concentration used in the standard test with organism. All the samples were kept in the same condition as during the test with *D. magna*. The test was performed in darkness in 20 °C. Samples were collected from each vessel within specific time intervals (after 0, 20, 24, 43 and 48 h) and analyzed using HPLC–DAD technique (parameters described in “Instrumental analysis”). Moreover, samples were divided into two groups: mix and not mixed before sampling. Each sample was prepared in duplicate.

### Baseline toxicity

In order to evaluate specific or non-specific mode of action, we used below equations (Escher and Hermens 2002):

1 $$ \log K\mathrm{mw}\kern0.5em =\kern0.5em 0.90\kern0.5em  \log K\mathrm{ow}\kern0.5em +\kern0.5em 0.521 $$

2 $$ \log \mathrm{EC}50\kern0.5em (M)\kern0.5em =\kern0.5em -0.77 \log K\mathrm{mw}\kern0.5em -\kern0.5em 1.89 $$

3 $$ \frac{\mathrm{EC}50\kern0.5em \left(\mathrm{baseline}\right)}{\mathrm{EC}50\kern0.5em \left(\mathrm{experimental}\right)}\kern0.5em =\kern0.5em \mathrm{TR} $$

where *K*_mw_ is membrane–water partition coefficient, *K*_ow_ is octanol–water partition coefficient, EC_50_—half maximal effective concentration, M—molar concentration and TR—toxic ratio.

## Results and discussion

### Instrumental analysis

Special emphasis should be placed on the importance of understanding the interplay between environmental chemistry and toxicology, thereby linking the concepts of bioavailability and the mechanism of ecotoxicity (Escher et al. 1997). Thus, combining chemical analysis with ecotoxicological tests can results with obtaining valuable data.

For this purpose, fully optimized and validated method for the determination of FEN and FLU has been applied. This was validated using working calibration standard solutions according to the procedures described in our previous studies (Migowska et al. 2012). The obtained validation parameters were as follows: the accuracy for FLU 95.3–102.5 % and for FEN 97.0–106.1 %, the precision described by relative standard deviation (RSD) was for FLU between 1.5 and 5.1 % and for FEN 1.2 and 4.6 %. The limit of quantification (LOQ) was 0.02 mg L^−1^ and the limit of detection (LOD) was 0.007 mg L^−1^ for both compounds. The correlation coefficient (*R* ^2^) amount to 0.9996 for FLU and 0.9987 for FEN.

#### Evaluation of soluble fraction of FEN and FLU in biological media

The deviation between nominal and real concentration of the test compounds in the media of our test systems has been tested via instrumental analysis to avoid misinterpretation of bioavailability and hence the obtained ecotoxicological data.

This aspect was crucial for FEN and FLU which have a low water solubility (Table 1). The standard stock solutions (500 mg L^−1^) prepared in DMSO were diluted in each biological media to concentrations mentioned in “Effect data modeling”. The addition of DMSO for *L. minor* and *S. vacuolatus* was 0.2 %, 0.06 % for *V. fischeri* and 0.015 % for FLU and 0.01 % for FEN for *D. magna*.

Nevertheless, this percentage of DMSO did not affect the organisms used in the tests, which was proved by testing the effect of control samples containing the same amount of DMSO in medium.

Results presenting the percentage of soluble fraction of FLU and FEN in biological media are presented in Table 2. These analyses indicate deviations (from −1.6 % for *D. magna* up to −24.3 % for *L. minor* for FLU and −4.4 % for *D. magna* up to 25.6 % for *S. vacuolatus* for FEN) between nominal and measured concentrations of stock solutions containing biological media. The nominal concentration for *D. magna* as well as for *V. fischeri* tests can be considered as the bioavailable fraction within the toxicity tests. However, results for *L. minor* and *S. vacuolatus* test medium would indicate underestimating the real toxicity if it would be exactly determined.

**Table 2:** Percentage of soluble fraction of examined compounds in biological media

Compound	Percentage of soluble fraction [%] (standard deviation)			
	*L. minor*	*S. vacuolatus*	*D. magna*	*V. fischeri*
FLU	75.7 (0.2)	78.2 (1.3)	98.4 (0.5)	96.1 (0.1)
FEN	75.0 (1.0)	74.4 (0.3)	95.6 (1.5)	83.0 (0.2)

### Chemical stability and adsorption studies

**Fig. 1:**
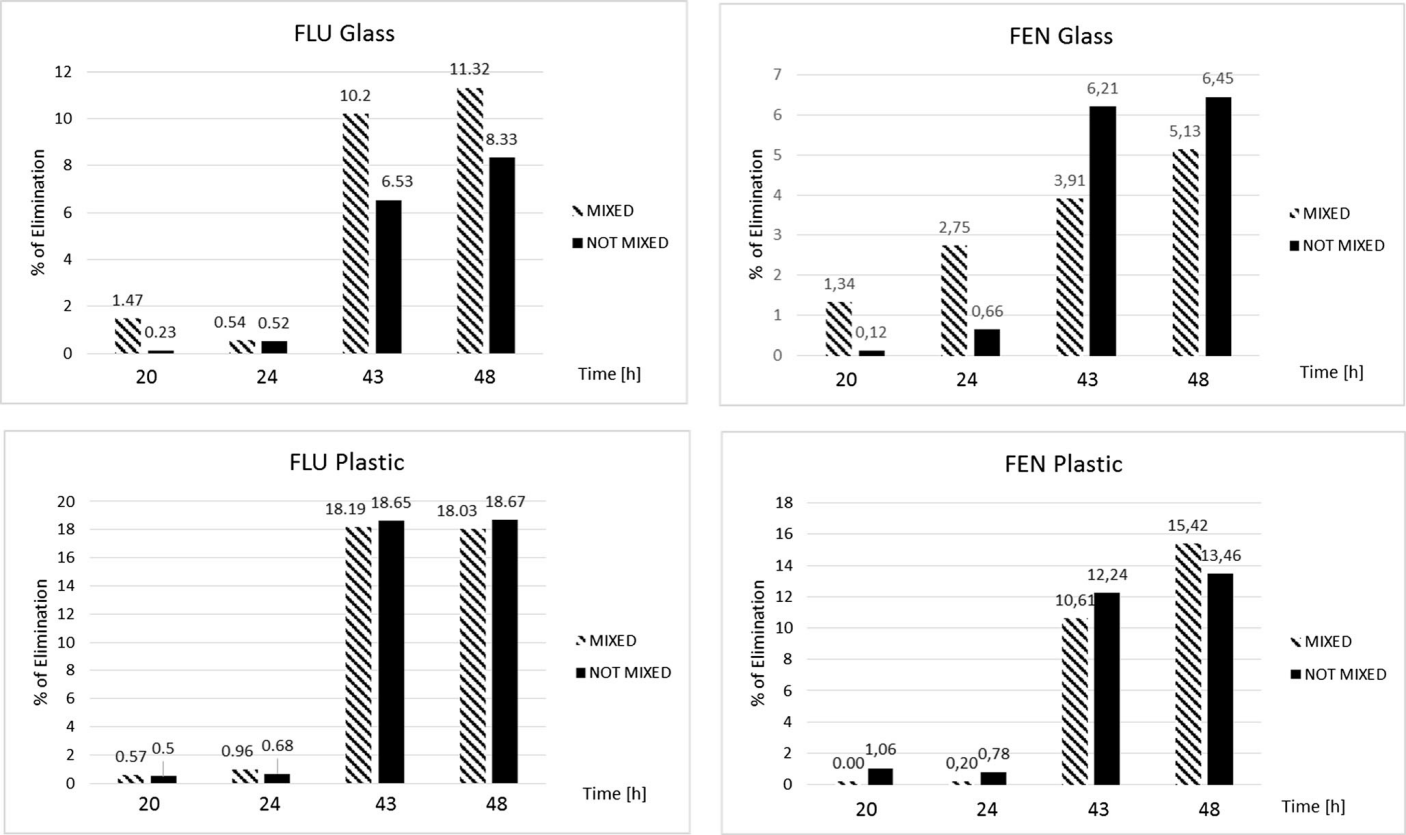
Results obtained during chemical and adsorptions studies (the percent of elimination is referring to the percet that has been eliminated from the solution)

Due to observed strong negative effect of the tested compounds to *D magna* (see “Toxicity tests”), additional stability and adsorption experiments were performed. The stability of FLU and FEN under the test conditions as well as possible adsorption of these drugs to plastic in comparison to the glass vessels was investigated over time (from time 0 to 48 h) to address two questions: (i) whether observed effects can be attributed to the parent compound or due to degradation products formed under test conditions; (ii) whether very narrow concentration range between which for all organisms effect is observed (effect 100 %) or no effect is observed (effect 0 %) is influenced by adsorption of these chemicals to the surface of the vessels used in the *D. magna* toxicity test. A decrease in concentration of the tested compounds could be proven in particular when polycarbonate vessel is used (Fig. 1). The mixing procedure before sampling was of minor importance. Since the elimination under such test conditions was low (maximal 11 %), the obtained ecotoxicological data have not been recalculated.

### Toxicity tests

In Table 3, EC_50_ values obtained in all ecotoxicity tests and for reference substances: atrazine and potassium dichromate are presented.

**Table 3:** The EC_50_ values for FLU and FEN obtained during experiments and toxicity of references compounds

Compound	EC_50_ (confidence interval) [mg L^−1^]			
	*D. magna*	*V. fischeri*	*L. minor*	*S. vacuolatus*
FLU	0.045 (0.043–0.046)	>0.3	>1	>1
FEN	0.019 (0.018–0.020)	>0.3	>1	>1
Atrazine	35.5^a^	69.4^a^	0.188^b^	0.039^c^
K_2_Cr_2_O_7_	0.6–2.1^d^	–	–	–

^a^Palma et al. 2008

^b^Teodorović et al. 2012

^c^Faust et al. 2001

^d^ISO Guideline 6341 1996

*D. magna* was the most sensitive aquatic organism towards investigated drugs (for FEN EC_50__48 h_ = 19 μg L^−1^ and for FLU EC_50 48 h_ = 45 μg L^−1^). These values are in agreement with Oh et al. (2006) who presented the EC_50 FEN_ = 16.5 μg L^−1^ and EC_50 FLU_ = 66.5 μg L^−1^ as well as with Hoechst-Roussel Agri-Vet (1995) who presented the EC_50_ only for FEN in the value of 12 μg L^−1^. No adverse effect on growth of algae *S. vacuolatus* and duckweed *L. minor* as well as to the luminescence of marine bacteria *V. fischeri* up to the highest tested concentration. Since already these concentrations exceed those found in the environment, we did not perform further experiments with the higher concentration range (Weiss et al. 2008; Van De Steene and Lambert 2008). The toxicity of FEN and FLU towards *L. minor* and algae *S. vacuolatus* is reported for the first time. However, such difference in sensitivity between algae and daphnids in acute toxicity tests was reported before for other group of pharmaceuticals, however also used as anthelmintic drug—abamectin (Tišler and Kožuh Eržen 2006). EC_50_ values obtained for algae *Scenedesmus subspicatus* (EC_50__72 h_ = 4.4 mg L^−1^) and *D. magna* (EC_50__48 h_ = 0.25 μg L^−1^) differed five orders of magnitude.

Also *V. fischeri* was much less sensitive to the studied benzimidazole anthelmintics than *D. magna*. It was reported that flubendazole and fenbendazole inhibited bacterial metabolism after 15 min of incubation by 50 % at similar concentrations 0.853 and 0.798 mg L^−1^ (Oh et al. 2006). These results are in agreement with our study since we did not observe any toxic effect to *V. fischeri* at concentration 0.3 mg L^−1^. Studies concerning their ecotoxicity to *V. fischeri* and *D. magna*, although are in agreement with the literature data, provide new insights, thanks to the results from the chemical analysis and prove high reliability of the existing and newly obtained data.

Apart from difference in species, sensitivity between the microbe and the invertebrate lower bioavailability of the tested benzimidazoles in the osmotically adjusted bacterial media was suggested as a possible cause of observed much lower toxicity of studied pharmaceuticals in *V. fischeri* in comparison to *D. magna*. This can be also an effect of other mode of toxic action of these pharmaceuticals in the selected organisms. However, chronic as well as mixture toxicity cannot be excluded (Wollenberger et al. 2000; Cleuvers 2003; Alexy et al. 2004); hence, further studies should be performed in the future.

High toxicity of FEN and FLU to *D. magna* was also reported by Oh et al. (2006). These authors explained these data by the fact that all tested benzimidazoles have a common chemical structure; therefore, the bioconcentration factor can be estimated by a quantitative structure–activity relationship model based on the octanol–water partition coefficient and the lipophilicity parameter (log *K*_ow_ of FEN = 3.93 (Mottier et al. 2003) and log *K*_ow_ of FLU = 2.91 (Horvat et al. 2012)) could explain most of the observed toxicity to *D. magna* of the benzimidazoles as well as the difference in their toxicity—FEN (EC_50_ = 19 μg L^−1^) occurred to be twice more toxic than FLU (EC_50_ = 45 μg L^−1^) .

Moreover, Escher highlights that about 60 % of all industrial chemicals act as baseline toxicants so that they interfere with the membrane structure and functioning simply by partitioning into the membrane (Escher 2001). Only certain compounds may additionally exhibit more specific and selective mechanisms. Therefore, in our study, we also conducted additional calculations to verify if FLU and FEN act as a baseline toxicants—whether they have specific or non-specific mode of action to non-target organisms. For this purpose, Eqs. 1, 2 and 3 (“Baseline toxicity”) were employed (Escher and Hermens 2002). Equation 1 (“Baseline toxicity”) refers to connection of log *K*_mw_ with log *K*_ow_ for polar compounds. Equation 2 is a QSARs of baseline toxicity based on *K*_mw_ as descriptor for *D. magna*. Equation 3 provides an information if the compound has specific mode of action or act as baseline toxicant (Escher et al. 2006). This parameter (TR) is the ratio of the predicted baseline effect concentration EC_50_ of a compound to the experimentally determined EC_50_. If TR is greater or equal than 10, it can be assumed that the mode of action is specific; if these parameter is less than 10, we are dealing with baseline toxicity. Data obtained in our investigation were as follows: TR_FLU_ = 341, TR_FEN_ = 153.

Since pharmaceuticals, as biologically active compounds, are designed to interact with a target molecule in the animal, in the environment they may affect other organisms having the same target or exert toxicity via other mechanisms (Gunnarsson et al. 2008). Different modes of benzimidazole action were reported. In studies with the parasite *Trichuris globulosa*, the anthelmintic effects of the benzimidazoles thiabendazole and fenbendazole were related to microtubule related process which is an inhibition of glucose uptake with resultant alterations in glucose metabolism (Jasra et al. 1990). Inhibition of amino peptidase activity and glutamate catabolism and increase in intracellular calcium levels were observed in *Echinococcus granulosus* protoscoleces after exposure to flubendazole (Cumino et al. 2009). In the in vitro studies with leukemia and myeloma cell lines, flubendazole induced cell death through mechanism related to alteration of microtubule structure and inhibited tubulin polymerization but, in contrast to the observation in parasite, alteration in glucose uptake was not observed (Spagnuolo et al. 2010). Moreover, at the genetic level changes in gene expression after 4 h of flubendazole treatment were observed, 196 genes were identified to be deregulated more than fourfold and 58 fell within eight functional annotations associated with chromosomal segregation and cytoskeleton regulation. Inhibition of tubulin polymerization can inhibit cell-cycle progression and induce mitotic catastrophe. It has been shown that flubendazole arrested cells in the G2 phase of the cell cycle and increased the number of multinucleated cells. On the basis of the performed experiments, it is hard to say which mechanism of action of the benzimidazoles caused adverse effects in test organisms and explain differences in their sensitivity to the studied pharmaceuticals. Since tubulin is found in both animal, plant and bacterial cells, it cannot be simply consistent with their effect as microtubule inhibitor. Hence, further more detailed studies are necessary.

## Conclusions

As a result of our research, we have presented a comprehensive study revealing the ecotoxicity of two benzimidazoles (fenbendazole and flubendazole) to four different organisms, from which toxicity to duckweed (*L. minor*) and green algae (*S. vacuolatus*) has been tested for the first time. Studies concerning their ecotoxicity to *V. fischeri* and *D. magna*, although are in agreement with literature data, provide new insights thanks to the results from the chemical analysis and prove high reliability of the existing and newly obtained data. The most sensitive organism among the tested ones was *D. magna* (the EC_50__48 h_ values for FLU and FEN were 45 μg L^−1^ and 19 μg L^−1^, respectively). As the presence of the investigated drugs in the environment was confirmed in concentrations in the range of few nanogram per liter up to microgram per liter (Weiss et al. 2008; Van De Steene and Lambert 2008; Zrnčić et al. 2014), their toxicity to *D. magna* at the levels of microgram per liter might influence these organisms in the ecosystem. However, further investigation should be taken into consideration concerning chronic tests in longer period of time. Moreover, we have performed additional calculations and included a discussion focused on a possible explanation of the observed strong ecotoxicity (exhibiting a specific MoA) of these drugs to *D. magna*.
